# Deguelin inhibits non-small cell lung cancer via down-regulating Hexokinases II-mediated glycolysis

**DOI:** 10.18632/oncotarget.15937

**Published:** 2017-03-06

**Authors:** Wei Li, Feng Gao, Xiaoqian Ma, Ruike Wang, Xin Dong, Wei Wang

**Affiliations:** ^1^ Department of Radiology, The Third Xiangya Hospital of Central South University, Changsha, Hunan, 410000, P.R. China; ^2^ Cell Transplantation and Gene Therapy Institute, The 3rd Xiangya Hospital of Central South University, Changsha, Hunan, 410000, P.R. China; ^3^ Powder Metallurgy Research Institute of Central South University, Changsha, Hunan, 41000, P.R. China; ^4^ Department of Ultrasonography, The 3rd Xiangya Hospital of Central South University, Changsha, Hunan, 410000, P.R. China; ^5^ Xiangya School of Medicine, Central South University, Changsha, Hunan, 410000, P.R.China; ^6^ State Key Laboratory of Molecular Oncology, Cancer Institute and Hospital, Chinese Academy of Medical Sciences and Peking Union Medical College, Beijing, 100000, P.R. China

**Keywords:** non-small cell lung cancer, deguelin, Hexokinases II, Akt, glycolysis

## Abstract

Hexokinases II (HK2) is a hub in the regulation of cancer cell glycolysis. Here we reported deguelin, a natural compound which has been studied in various tumor types, has a profound anti-tumor effect on human non-small cell lung cancer (NSCLC) via directly down-regulating of glycolysis. In NSCLC cell lines and primary NSCLC tissue, we found HK2 is overexpressed. Deguelin treatment markedly inhibited anchorage-dependent and independent growth of NSCLC cell lines. We revealed that deguelin exposure impaired glucose metabolism by inhibiting Akt-mediated Hexokinase II expression, overexpression of constitutively activated Akt1 substantially rescued deguelin-induced glycolysis suppression. Moreover, deguelin suppressed HK2 presence on mitochondrial outer membrane and induced apoptosis. The *in vivo* data indicated that deguelin prominently restrained tumor development in a xenograft mouse model. Thus, deguelin appears to be a promising new therapeutic agent for lung cancer and may be considered for further studies in other animal models and in clinical trials.

## INTRODUCTION

Non-small cell lung cancer (NSCLC) is the leading cause of cancer related-deaths worldwide [1, 2]. Epidemiological and laboratory animal model studies have demonstrated that host genetic susceptibility, smoking, and environmental exposure to carcinogens are closely linked to increased NSCLC risk [3, 4]. Despite advances in early detection and standard treatment, NSCLC is often diagnosed at an advanced stage and has a poor prognosis. The overall 5-year survival rate for NSCLC is less than 15%, which has hardly improved during the past few decades [5–7]. Changes in certain genes, such as mutation/inactivation of tumor suppressor genes (p53, LKB1, PTEN, et. al), or/and constitutively activation/amplification of oncogenes (EGFR, KRAS and PIK3CA, et. al), have been proved to associate with lung tumorgenesis [3, 8]. Although tyrosine kinase inhibitors (TKIs) and other targeted therapy drugs have shown an immediate and dramatic clinical response in patients with NSCLC that harbor somatic mutations in certain genes, most patients eventually develop acquired resistance [9, 10]. Therefore, there is an urgent demand to identify novel chemical entity with activity against NSCLC or develop novel therapeutic targets that can complement current chemotherapy.

In order to sustain unlimited growth, most cancer cells preferentially take glycolysis as their major energy source to rapidly generate ATP even in the presence of oxygen. This phenomenon, called the Warburg effect, is an emerging hallmark of cancer [11]. The hyperactivation of glycolysis induces greater nutrient uptake and an increased macromolecular biosynthesis for cancer cells. Additionally, the products of glycolysis, such as lactate, also provide an appropriate microenvironment to promote tumorigenic properties and tumor metastasis [12–14]. Hexokinase, which catalyzes the conversion of glucose to glucose-6-phosphate, is the first rate-limiting enzyme in glycolysis. Four distinct hexokinase isoforms, which is designated as hexokinase I-IV, have been identified in mammalian. However, only Hexokinases II (HK2) is predominantly expressed in malignant tumors and are critical for maintaining an elevated rate of aerobic glycolysis in cancer cells [15–18]. Overexpression of hexokinase-2, which was detected in diverse cancers, including lung, liver, breast, ovarian, gastric cancers and esophageal adenocarcinoma, not only compensates for the increased energy demands of cancer cells, but also confers tolerance to chemotherapy/radiotherapy and apoptosis resistance [16, 19–21]. Strikingly, clinical retrospective analysis disclosed that high expression of HK2 in tumor tissue indicated poor prognosis [22, 23]. This evidence suggests that inhibition of both HK2 and HK2-mediated glycolysis dysfunction might improve efficacy for cancer treatment.

In the present study, we found that deguelin, a natural compound isolated from the African plant *Mundulea sericea* (Leguminosae), has a potential inhibition effect on NSCLC both *in vitro* and *in vivo*. We demonstrated primarily that deguelin decreased HK2 and glycolysis in NSCLC and that Akt signaling pathway was confirmed to be crucial for deguelin-mediated HK2 expression and glycolysis regulation. These findings suggest that targeting metabolic enzymes, as well as the critical signing pathways, could be a new option for clinical NSCLC treatment.

## RESULTS

### HK2 is overexpressed in human non-small-cell lung cancer

**Figure 1:**
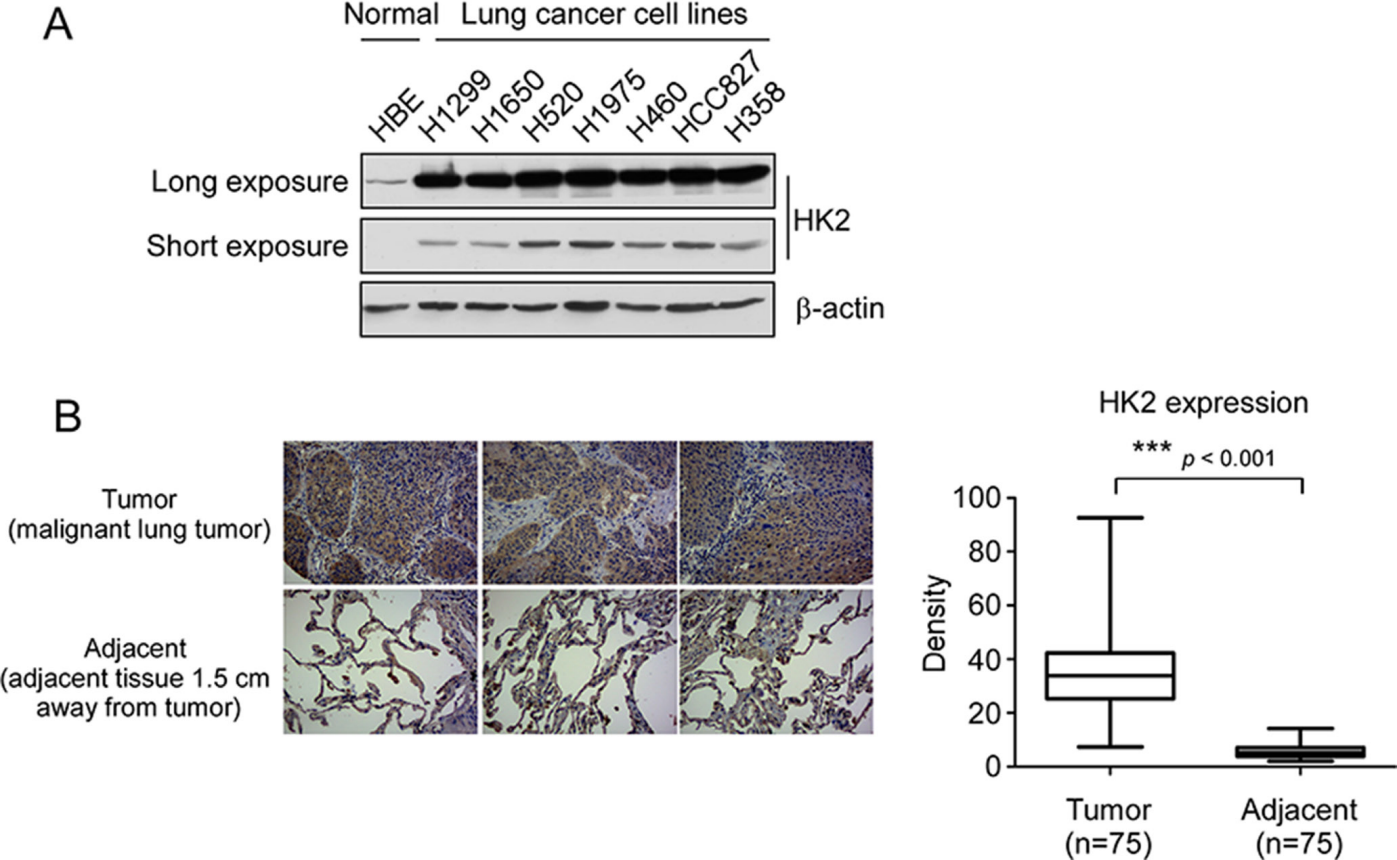
HK2 is overexpressed in lung cancer (**A**) HK2 is highly expressed in NSCLC cells. Western blot analysis was performed to examine HK2 expression in lung cancer cells and normal HBE lung cell, long time exposure, 5 min, short time exposure, 20 sec. (**B**) HK2 is highly expressed in lung cancer tissues. Immunohistochemical staining was performed on a lung cancer tissue array using a HK2 antibody. The intensity was evaluated using Image-Pro PLUS (v.6) and Image J (NIH) computer software. Statistical analyses were performed using Prism 5.0. The density of each individual sample is shown (right). The asterisk indicates a significant difference (***p < 0.001, Mann-Whitney *U*-test) between tumor and adjacent tissue as indicated.

The result from laboratory transgenic mice model study has demonstrated that HK2 is required for tumor initiation and maintenance in KRas-driven lung cancer [24]. To verify the high expression of HK2 in NSCLC, we determined the protein level of HK2 in different NSCLC cell lines and in a human NSCLC tissue array. Both long (long time exposure, 5 min) and short (short time exposure, 20 sec) time exposure data showed that, compared with the normal HBE cell, HK2 is highly expressed in NSCLC cell lines (Figure 1A). Immunohistochemistry data also demonstrated that HK2 is overexpressed in NSCLC tissues (Figure 1B). Furthermore, we found that knockdown of HK2 in H460 and H1650 cells inhibited both anchorage-dependent and –independent cell growth (Supplementary Figure 1). These results indicate that HK2 might be a critical molecule in NSCLC development.

### Deguelin suppresses proliferation and anchorage-independent growth of NSCLC cells

**Figure 2:**
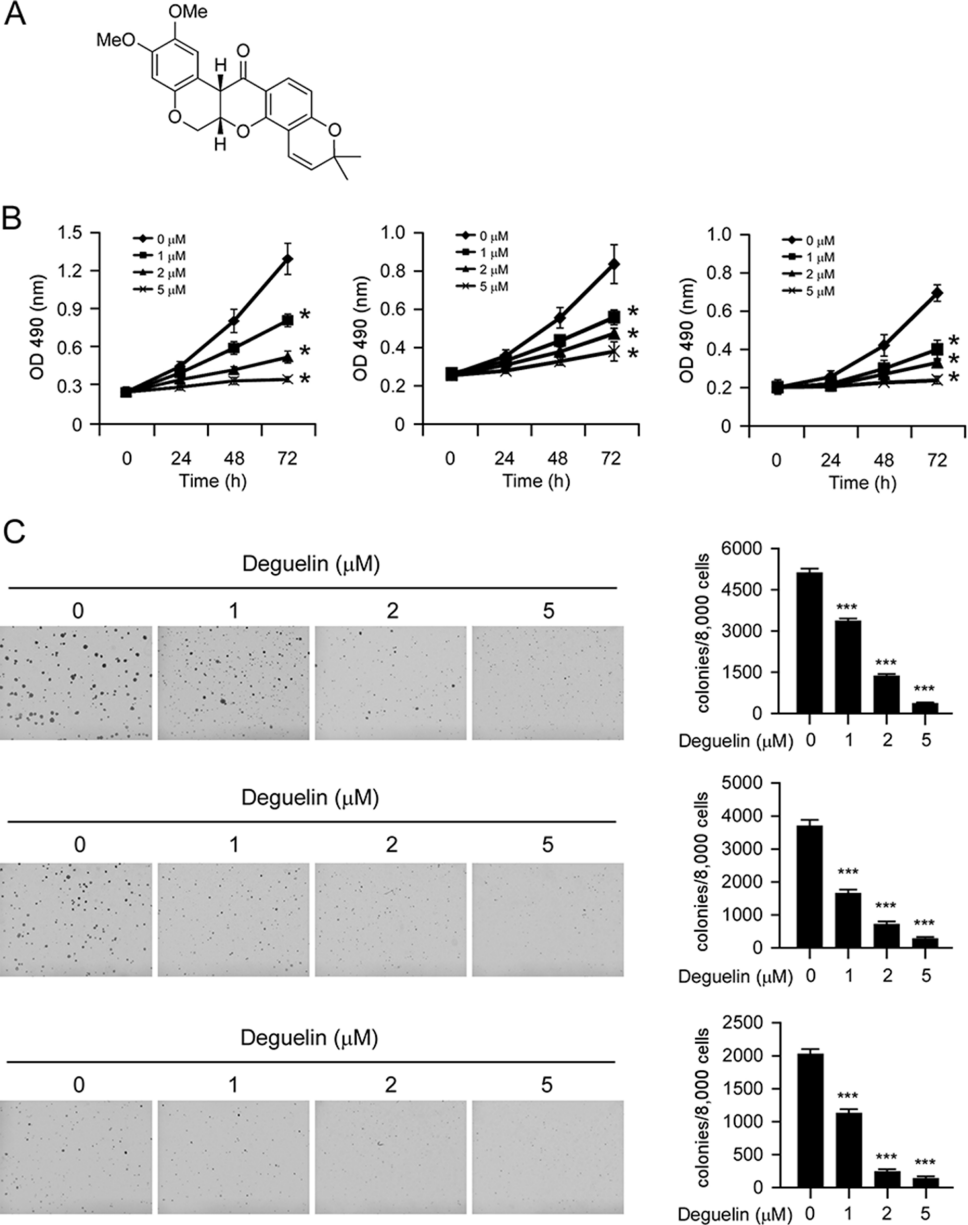
Inhibitory effects of deguelin on growth of NSCLC cells (**A**) Chemical structure of deguelin. (**B**) Deguelin inhibits anchorage-dependent growth in a panel of human lung cancer cells, including H460 (left), H1650 (middle) and HCC827 (right). Cell proliferation assay was performed as described in the “Material and Methods” section. Data shown are the proliferation ability of human NSCLC cells treated with different concentrations of deguelin compared with the dimethyl sulfoxide-treated group, asterisk, significant suppression (*p < 0.05) of proliferation by deguelin compared with DMSO treated group. (**C**) A colony formation assay was performed as described in the “Material and Methods” section. Data shown are the colony formation ability of H460 (up), H1650 (middle) and HCC827 (bottom) cells treated with different concentrations of deguelin compared with the dimethyl sulfoxide-treated group. The average colony number was calculated from three separate experiments. Asterisk, significant suppression (*p < 0.05, **p < 0.01, ***p < 0.001) of colony formation by deguelin compared with DMSO treated group.

Deguelin (Figure 2A, MW. 394.42) has shown potential chemopreventive activities against several types of human cancers. Our results indicated that the proliferation of human NSCLC cells, including H460 (Figure 2B, left), H1650 (Figure 2B, middle) and HCC827 (Figure 2B, right) were inhibited by deguelin in a dose-dependent manner (Figure 2B). Next, we examined the effect of deguelin on anchorage-independent growth of H460 (Figure 2C, top), H1650 (Figure 2C, middle), and HCC827 (Figure 2C, bottom) cells. As the results shown in Figure 2C, deguelin significantly suppressed the anchorage-independent growth of NSCLC cells at 1 μM, and 5 μM deguelin almost blocked the colony formation of NSCLC cells in soft agar. These results indicate that deguelin inhibits the anchorage-dependent and -independent growth of NSCLC cells in a dose-dependent manner.

### Deguelin down-regulates HK2 expression and glycolysis in NSCLC cells

**Figure 3:**
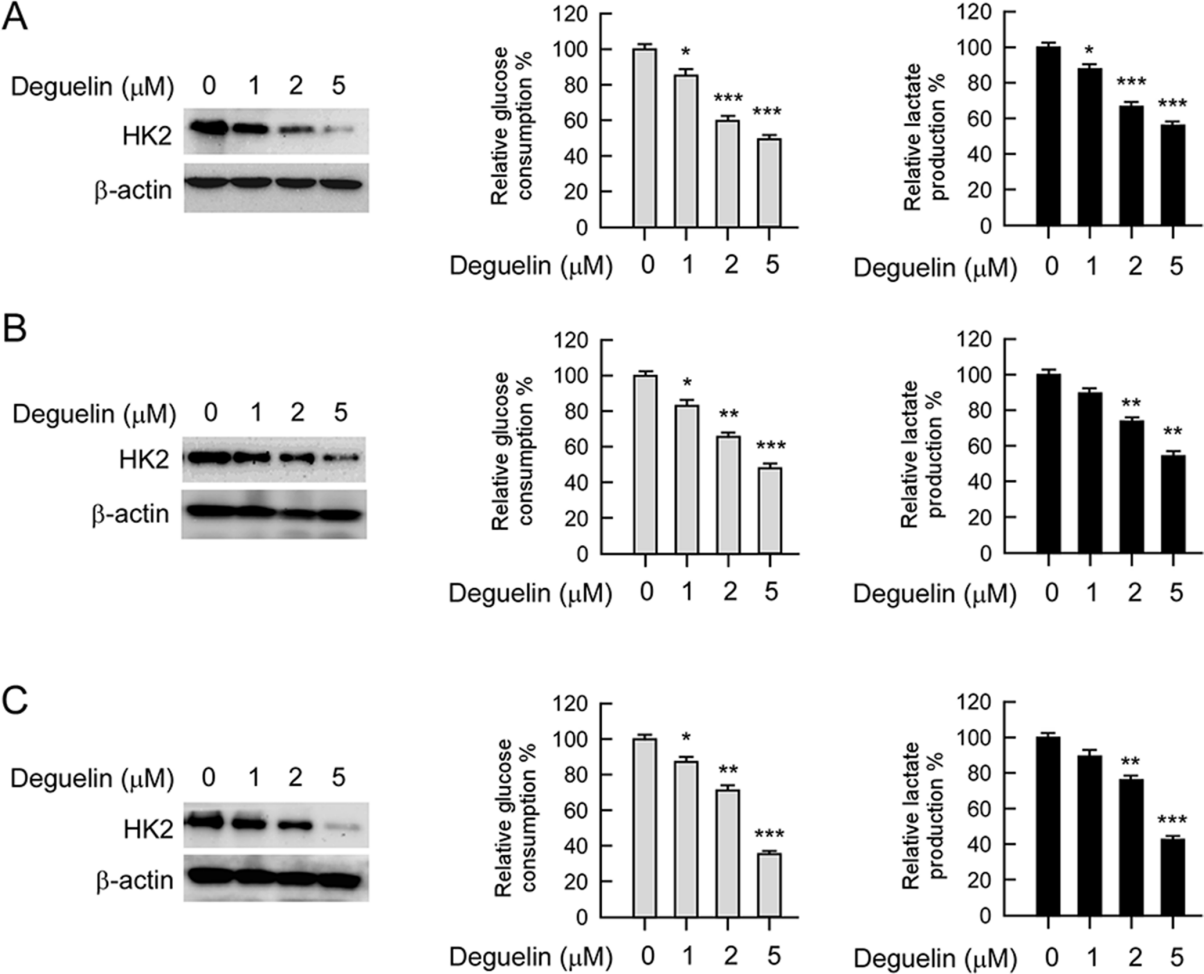
Deguelin regulates glycolysis in NSCLC cells NSCLC cells, including H460 (**A**), HCC827 (**B**) and H1650 (**C**) were treated with different concentrations of deguelin for 24 h. Western blotting was performed to detect HK2 expression (left). The levels of glucose consumption (middle) and lactate production (right) were examined in these cells. The asterisk, significant suppression (*p < 0.05, **p < 0.01, ***p < 0.001) of glycolysis by deguelin compared with DMSO treated group.

HK2, a rate-limiting enzyme of glycolysis, which is involved in cell growth regulation, is upregulated in multiple cancers. Based on the previous data that HK2 was overexpressed in human NSCLC and deguelin inhibited NSCLC cells growth, we then determined whether deguelin had any effect on HK2 expression and glycolysis in human NSCLC cells. The western blot data indicated that the HK2 total protein level was suppressed upon deguelin treatment (Figure 3, left). Consistent with the inhibition of HK2 expression, the glucose uptake (Figure 3, middle) and lactate production (Figure 3, right) of NSCLC cells were markedly inhibited after deguelin treatment.

H1650 (Figure 3A) cells treated with deguelin (1 μM) showed significantly lower glucose uptake than the untreated control. In H460 (Figure 3B) and HCC827 (Figure 3C) cells, deguelin also inhibited glucose consumption in a dose-dependent manner. With the reduction of glucose consumption, lactate production was also dramatically decreased after deguelin treatment. These results suggest that deguelin dose-dependently suppresses glycolysis in human NSCLC cells.

### Deguelin inhibits HK2 localization on mitochondrial outer membrane and induces apoptosis

**Figure 4:**
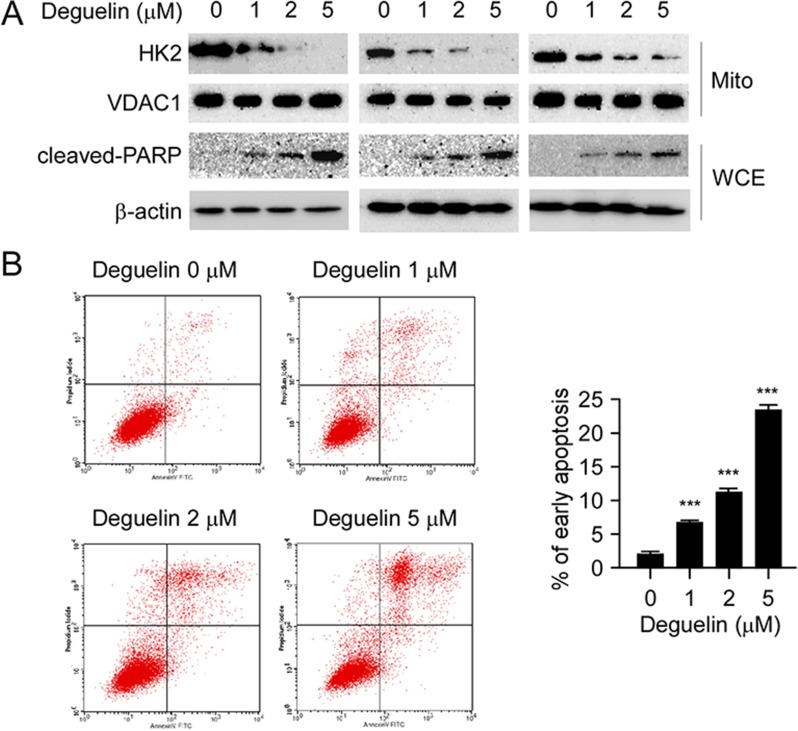
Deguelin suppresses HK2 localization on mitochondria and induces apoptosis (**A**) deguelin inhibits HK2 localization on mitochondria and up-regulates cleaved-PARP. H460 (left), HCC827 (middle) and H1650 (right) cells were treated with deguelin for 24 h as indicated, the mitochondrial fractions and whole cell extracts were isolated, western blot was conducted to detect target proteins. (**B**) deguelin induces apoptosis. H460 cells were treated with deguelin at the indicated concentrations for 24 h, flow cytometry was conducted as described in the “Material and Methods” section. Each experiment was conducted 3 times independently and the data are shown as means ± S.D., the asterisks indicate a significant increase (***p < 0.001) of deguelin induced apoptosis.

In cancer cells, HK2 overexpression is not only related to hyperactivated glycolysis but also associated with apoptosis resistance [15]. HK2 translocates to the mitochondrial outer membrane and interacts with the voltage-dependent anion channel (VDAC) to block the release of cytochrome c, and eventually inhibits the apoptosis pathway [25–27]. Indeed, our western blot data demonstrated that the localization of HK2 on the mitochondrial outer membrane was decreased in H1650 (Figure 4A, left), H460 (Figure 4A, middle) and HCC827 (Figure 4A, right) cells after deguelin treatment, and exposure to 5 μM deguelin nearly blocked the localization of HK2 on the mitochondrial outer membrane in H1650 and H460 cells. Furthermore, the cleaved-PARP, a bio-marker of apoptosis, in all of these tested NSCLC cells was markedly increased in response to deguelin treatment (Figure 4A). As expected, the flow cytometry data also indicated that deguelin induced apoptosis in H460 cells in a dose-dependent manner (Figure 4B), compared with the untreated group, the ratio of apoptosis cells reached to 20%. Based on these data, we proposed that deguelin-mediated decrease of mitochondrial-associated HK2 was related to apoptosis upregulation.

### Overexpression of HK2 rescues deguelin-induced mitochondrial apoptosis

**Figure 5:**
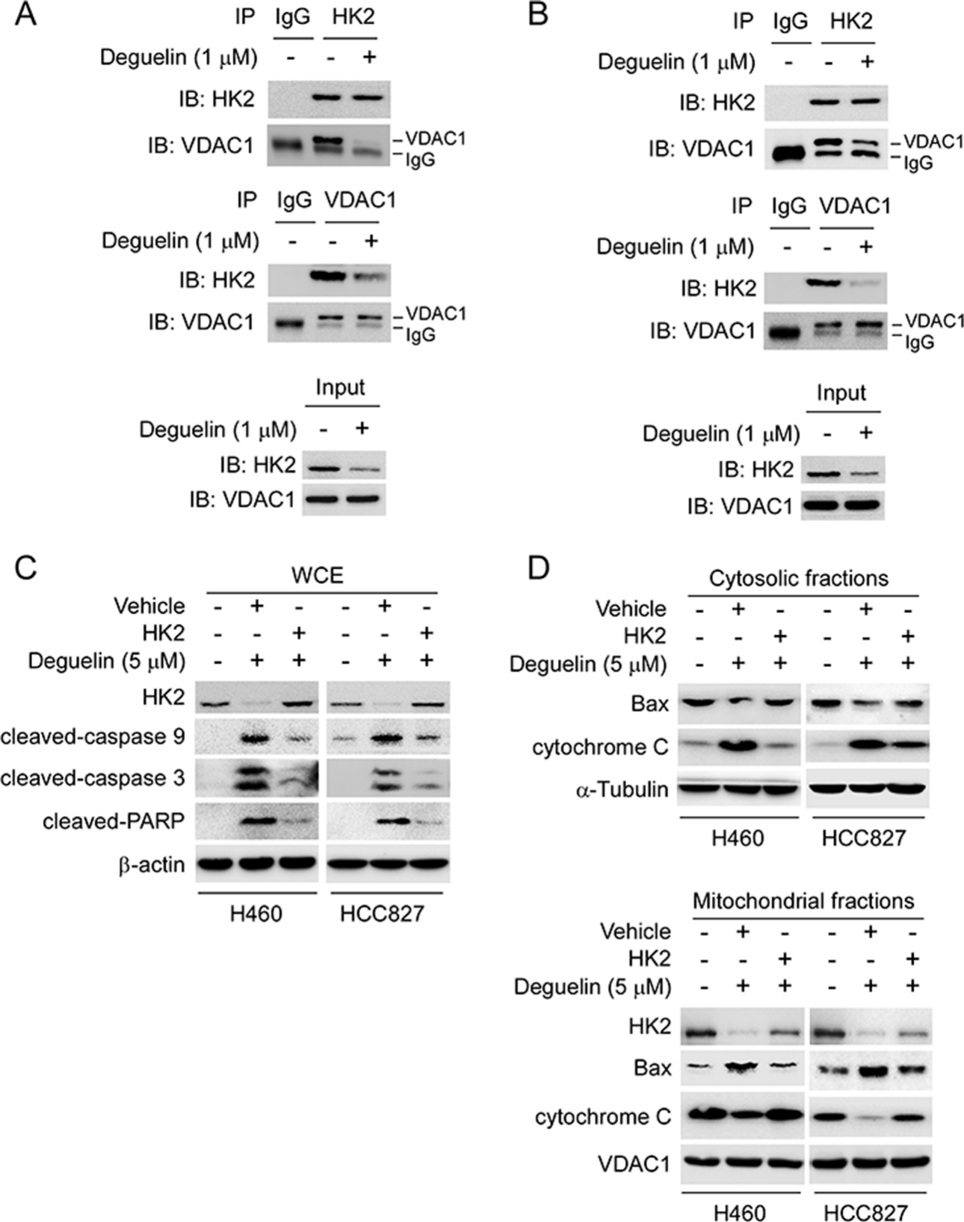
Overexpression of HK2 rescues deguelin-induced mitochondrial apoptosis (**A** and **B**) deguelin disrupts the interaction of HK2 and VDAC1. H460 (A) and HCC827 (B) cells were treated with deguelin for 24 h, co-immunoprecipitation was conducted to detect the interaction between HK2 and VDAC1. (**C** and **D**) overexpression of HK2 rescues deguelin-induced mitochondrial apoptosis in H460 cells. The HK2 or vehicle plasmid was transfected into H460 and HCC827 cells, after 24 h, these cells were treated with deguelin for another 24 h as indicated. Western blotting was performed to detect the protein expression in whole cell extraction (C) and subcellular fractions (D).

In order to further demonstrate that deguelin-induced apoptosis was related to HK2 mitochondrial localization and intrinsic apoptosis pathway, we studied the interaction between HK2 and VDAC1. Our results indicated that the endogenous HK2 and VDAC1 interaction in H460 (Figure 5A) and HCC827 (Figure 5B) cells were substantially down-regulated following deguelin treatment. In addition, overexpression of HK2 in H460 and HCC827 cells rescued deguelin-induced caspase9 and caspase3 activation, and reduced the expression of cleaved-PARP (Figure 5C). Upon apoptotic stimulation, Bax forms oligomers and translocates from the cytosol to the mitochondrial membrane. Indeed, as shown in the western blot data, we found that overexpression of HK2 inhibited deguelin-induced Bax mitochondrial localization and the release of cytochrome c from mitochondria (Figure 5D). Our results indicate that deguelin-induced mitochondrial apoptosis in NSCLC cells through the downregulation of HK2, which probably contributed to the activation and oligomerization of Bax on mitochondria.

### Akt is required for deguelin-mediated NSCLC cells glycolysis suppression

**Figure 6:**
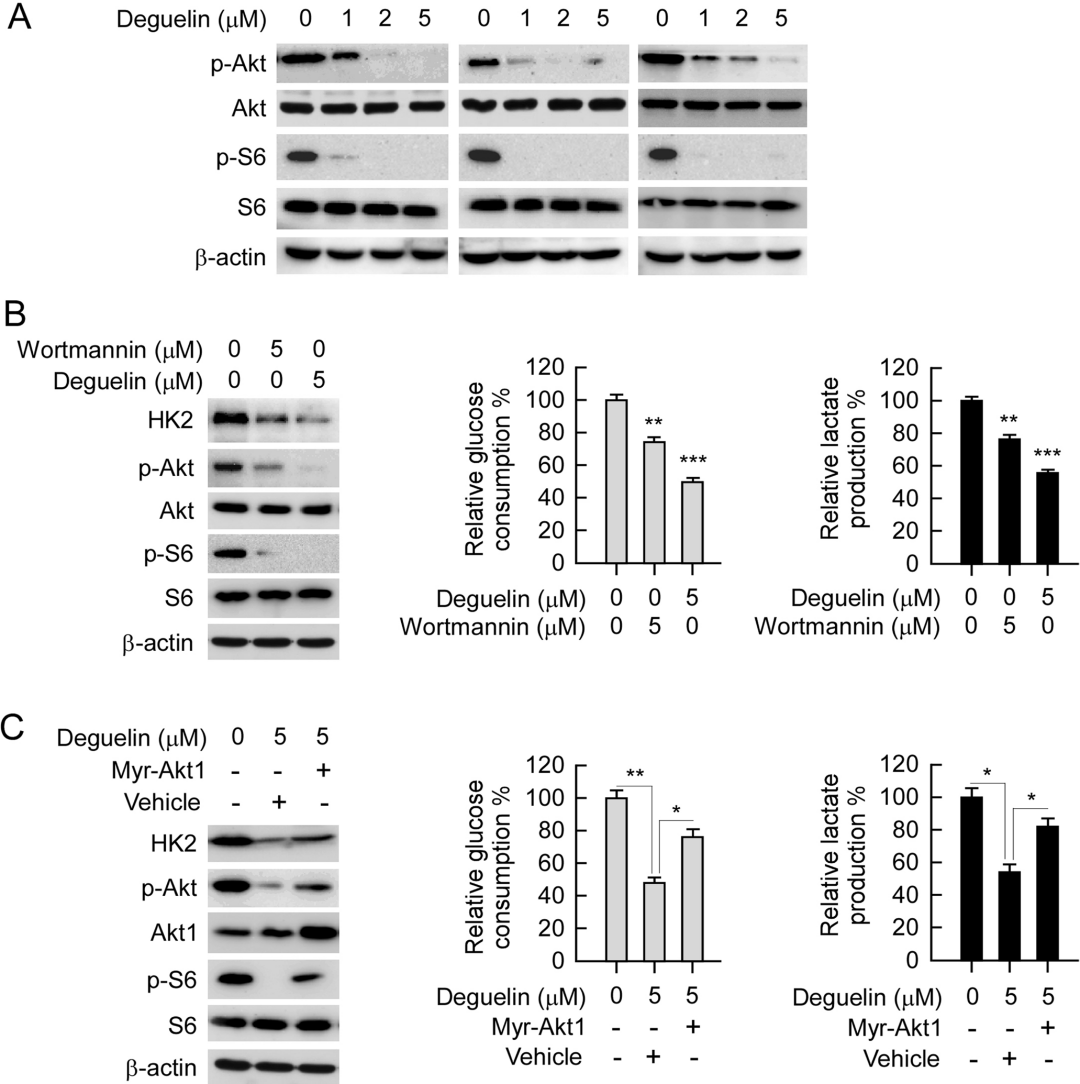
Akt is required for deguelin-suppressed NSCLC glycolysis (**A**) deguelin inhibits Akt signaling pathway. H460 (left), HCC827 (middle) and H1650 (right) cells were treated with deguelin for 24 h, western blot was conducted to detect target proteins as indicated. (**B**) deguelin, and wortmannin inhibit Akt activation and glycolysis. H460 cells were treated with deguelin or wortmannin for 24 h, protein levels were determined by western blotting (left), and glucose consumption (middle) and lactate production (right) were examined in these cells. (**C**) overexpression of constitutively active Akt (Myr-Akt1) rescues glycolysis in deguelin treated H460 cells. The Myr-Akt1 or vehicle plasmid was transfected into H460 cells, after 24 h, these cells were treated with deguelin for another 24 h as indicated. Western blot analysis was performed to detect the protein expression levels (left), and glucose consumption (middle) and lactate production (right) were examined in these cells. Asterisk, significant suppression (*p < 0.05, **p < 0.01, ***p < 0.001) of glycolysis between deguelin and DMSO treated group or Myr-Akt1 transfected group.

Akt is one of the most important kinases involved in HK2 expression regulation. Our data indicated that deguelin down-regulated Akt phosphorylation, as well as the phosphorylation of its downstream target S6 (Figure 6A), which is consistent with previous studies that deguelin is an efficient inhibitor for Akt activation [28, 29]. As expected, accompanied with the down-regulation of Akt activity, wortmannin and deguelin also induced the suppression of HK2 expression, as well as glycolysis in H460 cells (Figure 6B). In order to further elucidate the role of Akt played in deguelin-mediated glycolysis inhibition in NSCLC, constitutively activated Akt (Myr-Akt1) was transfected into H460 cell. As shown in Figure 6C, with the increase of Akt activity, deguelin-mediated decrease of HK2 was significantly attenuated in contrast with the vehicle group. This result further confirmed that Akt signaling pathway was involved in the regulation of HK2 expression in NSCLC cells. Along with the increase of HK2 expression in Myr-Akt1 transfected H460 cell, deguelin-induced suppression of glucose consumption and lactate production were significantly rescued (Figure 6C). The real-time PCR result indicated that deguelin-induced down-regulation of HK2 mRNA transcription was rescued by Myr-Akt1 overexpression (Supplementary Figure 2). Moreover, we found that Knockdown of Akt1 in H460 cells inhibited both HK2 expression and glycolysis (Supplementary Figure 3). The results imply that Akt inhibition is required for deguelin-mediated NSCLC cells glycolysis suppression.

### Deguelin suppresses xenograft tumor growth

**Figure 7:**
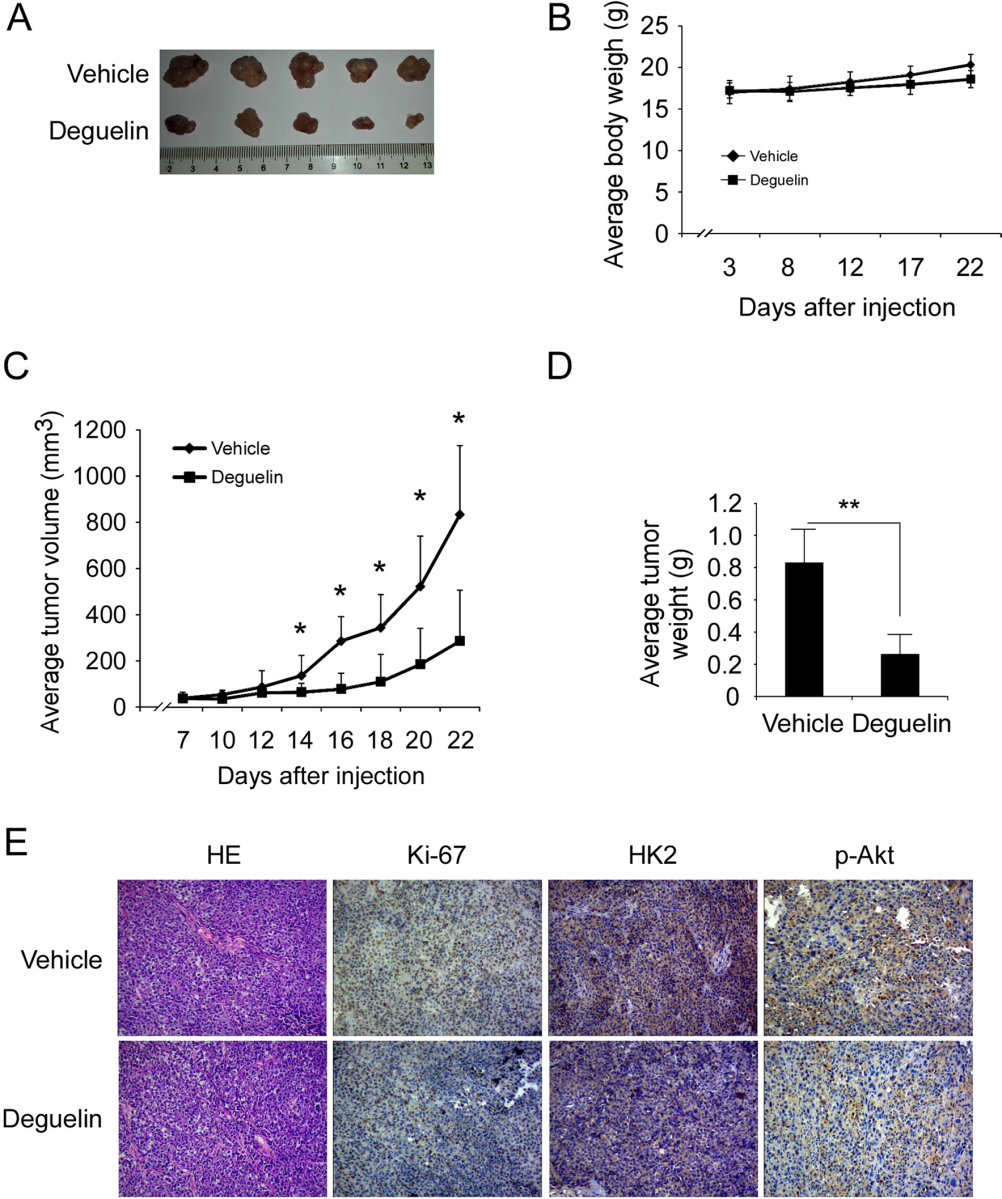
Deguelin inhibits tumor growth in xenograft mouse model (**A**) At the treatment end point, mice were sacrificed and tumors were removed, weighed and photographed. (**B**) The body weight of mice from the vehicle group and deguelin-treated group was measured. (**C**) The tumor volume of mice from the vehicle group and deguelin-treated group was measured. (**D**) The tumor weight of mice from the vehicle group and deguelin-treated group was measured. For B, C, and D, data are shown as mean values ± S.D. obtained from 5 mice in each group. (**E**) Immunohistochemical staining examination of Ki67, HK2 and p-Akt in tumor sections from vehicle group mice or deguelin-treated group mice. All panels are of the same magnification. The asterisks indicate a significant difference (* *p* < 0.05, **p < 0.01) between deguelin-treated group and vehicle group.

To determine the chemotherapeutic effect of deguelin *in vivo*, we used an athymic nude xenograft mouse model of H460 cell. Data showed that deguelin inhibited tumor growth in the H460 xenograft model as expected (Figure 7A, 7D), 22 days after tumor cell injection, the tumor volume of vehicle group had reached about 800 mm^3^, but in deguelin-treated group, the average tumor volume was around 300 mm^3^ (Figure 7C). Meanwhile, no obvious toxicity was observed as evaluating the change of body weight of tumor-bearing mice between vehicle and deguelin-treated group (Figure 7B). Immunohistochemical analysis revealed that the Ki-67, p-Akt and HK2 were significantly suppressed in the deguelin-treated group (Figure 7E). Additionally, the western blot and immunohistochemical staining results found that deguelin promoted H460 cells apoptosis *in vivo* (Supplementary Figure 4). These results clearly indicate that deguelin exerts a substantial chemotherapeutic effect to xenograft tumor growth in mice through the suppression of Akt activity and HK2 expression.

## DISCUSSION

An accumulation of evidence reveals that deguelin, a natural product, acts as a novel anti-tumorigenic agent targeting apoptosis, cell cycle arrest, and anti-angiogenesis for cancer chemoprevention and chemotherapy in various types of cancer both *in vitro* and *in vivo* [30–33]. Although the suppression of multiple protein kinases, transcription factors and signal transduction pathways are involved in deguelin-mediated anti-tumor activities, the underlying mechanisms of how deguelin utilizes the tumor suppression effect through the metabolic pathways, were not fully understood [30]. Here, for the first time, we demonstrated that along with the down-regulation of HK2, the glycolysis in NSCLC was significantly inhibited after deguelin treatment. Deguelin decreased HK2 expression and inhibited HK2 localized onto the mitochondrial outer membrane. We disclosed that the inhibition of glycolysis, caused by deguelin in NSCLC, was closely linked to deguelin-mediated Akt activity suppression.

For cancer cells to sustain their rapid proliferation and gain a survival advantage, glycolysis has been demonstrated as a hot spot for metabolic reprogramming. The hyperactivation of glycolysis not only generates ATP and intermediates for cancer cells growth, but also induces tumor progression [11, 34, 35]. HK2 plays a pivotal role in the process of tumor glycolysis regulation. Numerous and compelling data from laboratory and clinical investigations has revealed that HK2 was overexpressed in various types of human cancers, including NSCLC [36–38]. We confirmed through this study that HK2 is overexpressed in NSCLC cell lines and lung cancer tissues (Figure 1), which implied that HK2 may has a crucial role during lung tumorgenesis. Recently, some natural compounds, such as resveratrol [39] and EGCG [40, 41], have been demonstrated to inhibit hepatocellular carcinoma or oral cancer via directly targeting glycolysis. These findings implied that decreases of glycolysis or inhibits of metabolic enzymes may act as one of the major mechanisms in natural compound mediated anti-tumor activities. Indeed, accompanied with the down-regulation of HK2, deguelin also significantly induced glucose consumption and lactate production suppression (Figure 3). It is widely accepted that overexpression of HK2 in cancer cells is associated with chemotherapy tolerance and apoptosis resistance. Co-targeting HK2-mediated Warburg effect and ULK1-dependent autophagy suppresses tumor growth of PTEN- and TP53-deficiency-driven castration-resistant prostate cancer [42]. HK2 can translocate to the mitochondrial outer membrane and interacts with the pore-like protein voltage-dependent anion channel (VDAC) to form a complex to manipulate the release of cytochrome c, and eventually inhibiting apoptosis [15, 26, 27]. Xiao *et al* reported that targeting Epstein–Barr virus oncoprotein latent membrane protein 1 (LMP1)-mediated-glycolysis and -HK2 sensitizes nasopharyngeal carcinoma to radiation therapy [43]. Recently, Martin *et al* indicated that deprivation of androgen leads to increased carbohydrate metabolism and HK2-mediated survival in Pten/Tp53-deficient prostate cancer [44]. This evidence suggested that HK2 or the glycolysis signaling pathway remains a promising target of anti-cancer therapy. Consistent with our results, with the decrease of HK2, the interaction between HK2 and VDAC was weakened, which resulted in the change of permeability of mitochondrial membrane and cell apoptosis. More importantly, deguelin-induced NSCLC apoptosis was at least partially dependent on HK2 mitochondrial localization (Figure 5). Our data uncovered a previously unknown mechanism of deguelin-induced cancer cells death and implies that HK2 offers a candidate molecular target for NSCLC therapy.

The PI3K signaling pathway plays a very important role in both in NSCLC and Small Cell Lung Cancer. Approximately 4% of NSCLC patient samples were found to harbor a mutation in the *PIK3CA* gene. More importantly, the amplification of *PIK3CA* was detected in over 30% of squamous-cell carcinoma biopsy [3, 45]. The constitutive activation of PI3K/Akt signaling pathway confers tumorigenic properties of NSCLC, as well as drug resistance, recurrence and metastasis [6, 46–50]. Deguelin, a well-known PI3K/Akt pathway inhibitor, was first reported by Dr. Lee in 2003 [28]. In the past decade, the anti-tumor effect and the molecular targets of deguelin has been widely studied. Intriguingly, deguelin has shown to have potential anti-cancer or chemoprevention activities in lung cancer through various signaling pathways both *in vitro* and *in vivo* [28, 29, 51–53]. Similarly, our results demonstrated that deguelin dramatically inhibited Akt activation, the down-regulation of HK2, and glycolysis. This was shown after deguelin treatment was dependent on deguelin-mediated Akt activity suppression and when transient transfection of constitutively activated Akt1 rescued the impaired glycolysis in H460 cell (Figure 6). Even though the underlying mechanisms of the deregulation of HK2 in cancer cells have not yet been completely elucidated, the PI3K/Akt-related signaling pathway [54], as well as the transcription factors c-myc and hypoxia inducible factors-1α (HIF-1α) [55], has been demonstrated to be involved in HK2 and HK2-mediated glycolysis regulation. In human prostate cancer, deguelin treatment directly decreased c-Myc expression [56], which implied that in NSCLC, other molecular or signaling pathways may also involve in deguelin-induced HK2 or glycolysis suppression.

Briefly, the present study suggested that glycolysis is involved in deguelin-mediated anti-tumor activity. For the first time, deguelin against NSCLC through the glucose metabolic pathway was investigated *in vitro* and *in vivo*. We identified HK2, a downstream target of PI3K/Akt, as a new potential target of deguelin. Suppression of the PI3K/Akt-HK2 signaling pathway was proposed to be one of the major underlying mechanisms for deguelin to possess its anti-NSCLC effect in this story. The present study suggests that HK2 is a good molecular target for clinical treatment of NSCLC.

## MATERIALS AND METHODS

### Reagents and antibodies

Tris, NaCl, and SDS for molecular biology and buffer preparation and deguelin (> 99%) were purchased from Sigma (St. Louis, MO). Cell culture media and supplements were from Invitrogen (Grand Island, NY). Antibodies against HK2 (2867, IB: 1:2000, IHC:1:200), p-Akt (4060, IB: 1:1000, IHC: 1:100), Akt (4691, IB: 1:2000), Akt1 (75692, IB:1:2000), p-S6 (4858, IB: 1:4000), S6 (2317, IB:1:2000), VDAC1 (4866, IB:1:2000), cleaved-caspase 3 (9664, IB: 1:2000), cleaved-caspase 9 (9505, IB: 1:1000), cleaved-PARP (5625, IB: 1:2000) and cytochrome C (4280, IB: 1:1000) were obtained from Cell Signaling Technology, Inc. (Beverly, MA). Antibody against β-actin (A5316, IB: 1:10000) and Bax (B8429, IB: 1:2000) were from Sigma-Aldrich. Antibody against α-Tubulin (SC-5286, IB: 1:5000) was from Santa Cruz. Anti-Ki67 (ab16667, IHC: 1: 250) was purchased from Abcam (Cambridge, UK).

### Cell culture and transfection

Cells from American Type Culture Collection (ATCC, Manassas, VA) were cultured at 37°C in a humidified incubator with 5% CO_2_ according to ATCC protocols. Cells were cytogenetically tested and authenticated before being frozen. Each vial of frozen cells was thawed and maintained for 2 months (10 passages). Human NSCLC cells, including H460, H1650, H1299, H520, HCC827, H1975 and H358 were grown in RPMI-1640 medium supplemented with 10% FBS and antibiotics. HBE human bronchial epithelial cell was cultured with BEGM supplemented with 10% FBS and antibiotics. For transfection experiments, the jetPEI (Qbiogene, Inc., Montreal, Canada) transfection reagent was used following the manufacturer's instructions. The cells were cultured for 36–48 h and protein extracted for analysis.

### MTS assay

Human NSCLC cells were seeded (2 × 10^3^/well/100 ml) into 96-well plates, and proliferation was assessed by MTS assay (Promega, Madison, WI) according to instructions provided.

### Anchorage-independent growth

Human NSCLC cells were suspended (8,000 cells/ml) in 1 ml of 0.3% agar with Eagle's basal medium containing 10% FBS, 1% antibiotics, and different concentrations of deguelin overlaid into six-well plates containing a 0.6% agar base. The cultures were maintained in a 37°C, 5% CO_2_ incubator for 1 to 2 weeks, and then colonies were counted under a microscope using the Image-Pro Plus software program (Media Cybernetics, Silver Spring, MD).

### Glucose uptake and lactate production measurement

NSCLC cells were trypsinized and seed in 6-well plates (5 × 10^5^), after incubation for 24 h, medium was discarded and cells were incubated in fresh medium containing different concentrations of deguelin for 8 h. Glucose and lactate levels were measured (Automatic Biochemical Analyzer; 7170A, HITACHI, Tokyo, Japan) at the Clinical Biochemical Laboratory of Xiangya Hospital (Changsha, China). The relative glucose consumption rate and lactate production rate were normalized by the protein concentration of samples.

### Immunoblotting and immunoprecipitation

Protein samples were extracted with RIPA buffer (10 mM Tris-Cl (pH 8.0), 1 mM EDTA, 0.5 mM EGTA, 1% Triton X-100, 0.1% sodium deoxycholate, 0.1% SDS. 140 mM NaCl). For immunoblotting, protein samples (30 μg) were separated by 10% SDS-PAGE, transferred onto PVDF membranes and blocked with 5% non-fat milk for 1h at room temperature. The membranes were incubated with primary antibodies at 4°C overnight. Then, the membranes were washed with PBST for three times. Binding of primary antibodies was detected using peroxidase conjugated secondary antibodies (Cell Signaling Technology, Inc. Beverly, MA). Then, the membranes were washed with PBST for three times. Proteins were visualized with an enhanced chemiluminescence detection kit (Pierce ECL, Thermo Scientific, Pittsburgh, PA.). For immunoprecipitation, extracts were precleared with 30 mL (50% slurry) agarose A/G beads by rocking for 2 h at 4°C. Beads were removed and 30 mL (50% slurry) of fresh agarose A/G beads and appropriate antibodies were added to the precleared lysates overnight at 4°C. The beads were washed with wash buffer for three times, mixed with 6 ×SDS sample buffer, boiled, and then resolved by SDS–PAGE. Proteins were detected with specific antibodies and a peroxidase conjugated secondary antibody.

### Isolation of mitochondrial fractions

Following deguelin treatments, approximately 5 × 10^6^ cells from a 10 cm plate were harvested by trypsinization and centrifuged at 800 rpm for 5 min at 4°C. The cell pellets were washed once with ice cold PBS and then resuspended in 3 volumes of isolation buffer (20 mM Hepes, pH 7.4, 10 mM KCl, 1.5 mM MgCl2, 1 mM sodium EDTA, 1 mM dithiothreitol, 10 mM phenylmethylsulfonyl fluoride, 10 mM leupeptin and 10 mM aprotinin) in 250 mM sucrose. After chilling on ice for 3 min, the cells were disrupted by 60 strokes of a glass homogenizer. The homogenate was centrifuged once at 2,000 rpm at 4°C for 10 min to remove unbroken cells and nuclei. The mitochondria-enriched fraction (supernatant) was then pelleted by centrifugation at 13,000 rpm for 30 min. The pellets was lysed in RIPA buffer (10 mM Tris-Cl (pH 8.0), 1 mM EDTA, 0.5 mM EGTA, 1% Triton X-100, 0.1% sodium deoxycholate, 0.1% SDS. 140 mM NaCl) and analyzed by western blot.

### Flow cytometry

For apoptosis analysis, the cells were suspended in 1 × 10^6^ cells/ml, and 5 μl Annexin V and Propidium Iodide Staining Solution were added to 300 μl of the cell suspension. After incubated 10–15 min at room temperature in the dark, Stained cells were assayed and quantified using a FACSort Flow Cytometer (BD, San Jose, CA, USA).

### *In vivo* tumor growth

All the experimentation for animals was approved by the Animal Ethics Committee of Central South University (No: 2016-S000), Hunan Province, China. H460 human lung cancer cells (1 × 10^6^) in 100 μL 1640 medium were inoculated s.c. into the right flank of 5-week-old female athymic nude mice. Five days after inoculation, mice were given an i.p. injection of deguelin at a dose of 4 mg/kg every three days, whereas control mice were administered vehicle. The body weight of each mouse was recorded and tumor volume was determined by vernier caliper twice a week. Volume was calculated following the formula of A × B^2^ × 0.5, wherein A is the longest diameter of tumor, B is the shortest diameter and B^2^ is B squared.

### Immunohistochemical staining

A human NSCLC tissue array (Hlug-NSCLC150PT-01) from Shanghai Outdo Biotech Co., Itd. (Shanghai, China) and included 37 cases of adenocarcinoma, 30 cases of squamous cell carcinoma, 3 cases of large cell carcinoma, 5 cases of bronchioloalveolar carcinoma and 75 cases of matched adjacent normal tissue. A Vectastain Elite ABC Kit (Vector Laboratories; Burlingame, CA) was used for immunohistochemical staining according to the recommended protocol. Briefly, the tissue array and tissue sections form xenograft model were baked at 60°C for 2 h, deparaffinized, and rehydrated. To expose antigens, the slide was unmasked by submersion into boiling sodium citrate buffer (10 mM, pH 6.0) for 10 min, and then treated with 3% H_2_O_2_ for 10 min. The slide was blocked with 50% goat serum albumin in 1×PBS in a humidified chamber for 1 h at room temperature and then with a first antibody (1:100 dilution in 50% goat serum with PBS) at 4°C in a humidified chamber overnight. The slide was washed and hybridized with the secondary antibody from Vector Laboratories (Burlingame, CA) (anti-rabbit 1:200) for 1 h at room temperature. Slides were stained using the Vectastain Elite ABC kit. The intensity was estimated by Image-Pro PLUS (v.6) and Image J (NIH) software programs. Statistical analyses were performed using Prism 5.0.

### Statistical analysis

All quantitative data are expressed as mean values ± S.D of at least 3 independent experiments. Significant differences were determined by Student's *t* test or Mann-Whitney *U*-test. A probability value of *p* < 0.05 was used as the criterion for statistical significance.

## SUPPLEMENTARY MATERIALS FIGURES


