# Supplementary material for: Deguelin inhibits non-small cell lung cancer via down-regulating Hexokinases II-mediated glycolysis
Source: Oncotarget. 2017 Mar 6;8(20):32586–99. doi: 10.18632/oncotarget.15937 (PMC5464811; doi:10.18632/oncotarget.15937)
Supplement: Supplementary file 1 [file oncotarget-08-32586-s001.pdf]

Deguelin inhibits non-small cell lung cancer via down-regulating Hexokinases II-mediated glycolysis

Supplementary Materials

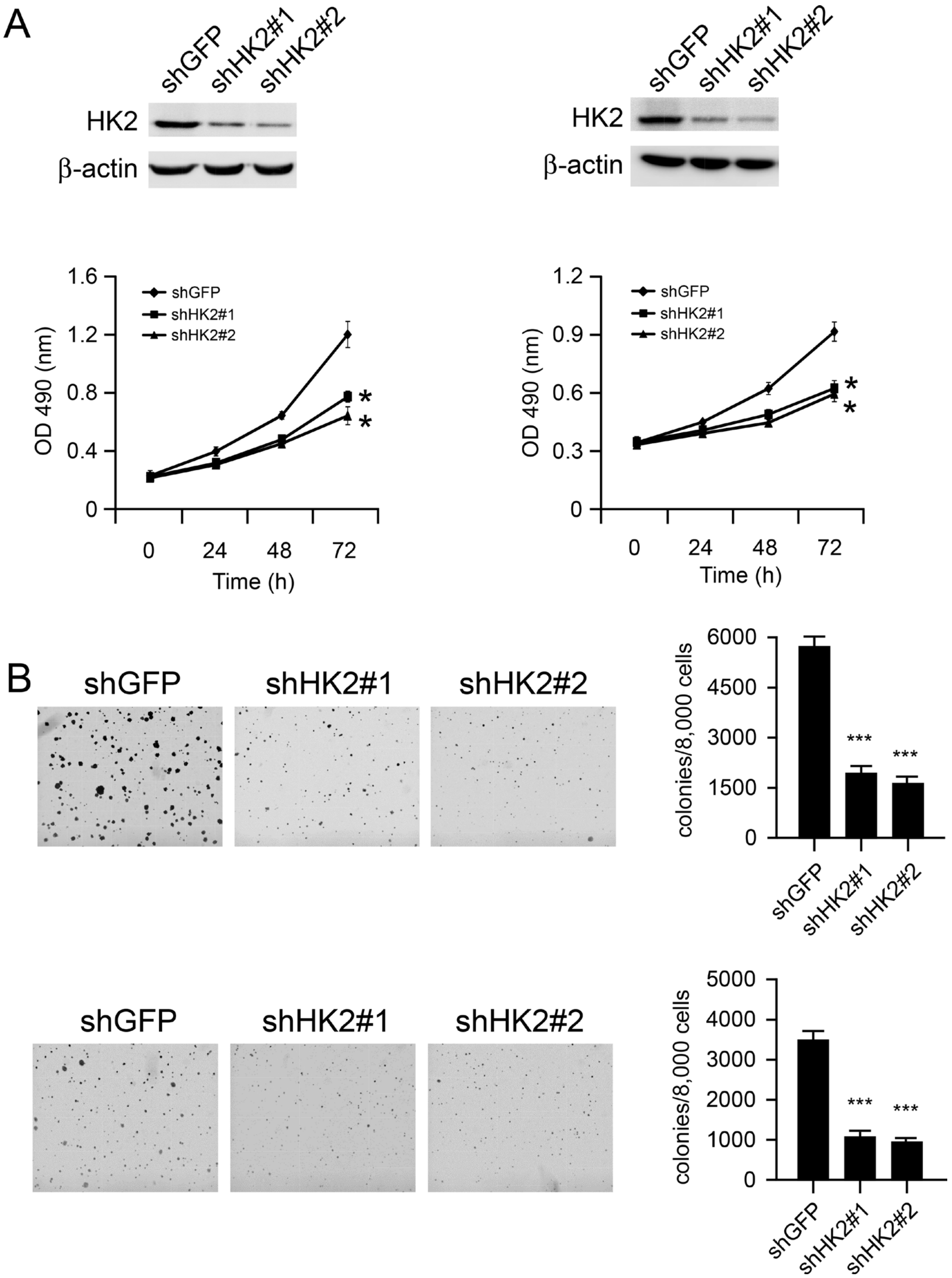

Supplementary Figure 1: Knockdown of HK2 inhibits tumor cell growth. (**A**) western blot verifies knockdown of HK2 in H460 (left) and H1650 (right) cells. (**B**) the growth curves show the difference in anchorage-dependent growth between GFP and HK2 knockdown cells, left, H460, right, H1650 (*$p < 0.05$). (**C**) soft agar assay show the difference in anchorage-independent growth between GFP and HK2 knockdown cells, top, H460, bottom, H1650. Representative photographs are shown, and the graph (right) shows data from at least 3 independent experiments expressed as means ± S.D. The asterisk (***$p < 0.001$) indicates a significant decrease in colony formation by knockdown cells.

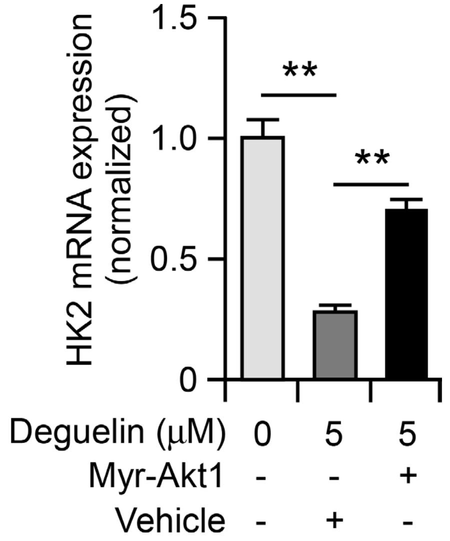

Supplementary Figure 2: Real-time RT-PCR analysis of total HK2 mRNA levels in H460 cells. H460 cells were treated with deguelin as indicated, the mRNA was extracted for Real-time PCR analysis. The data are expressed as means ± SD (**p < 0.01).

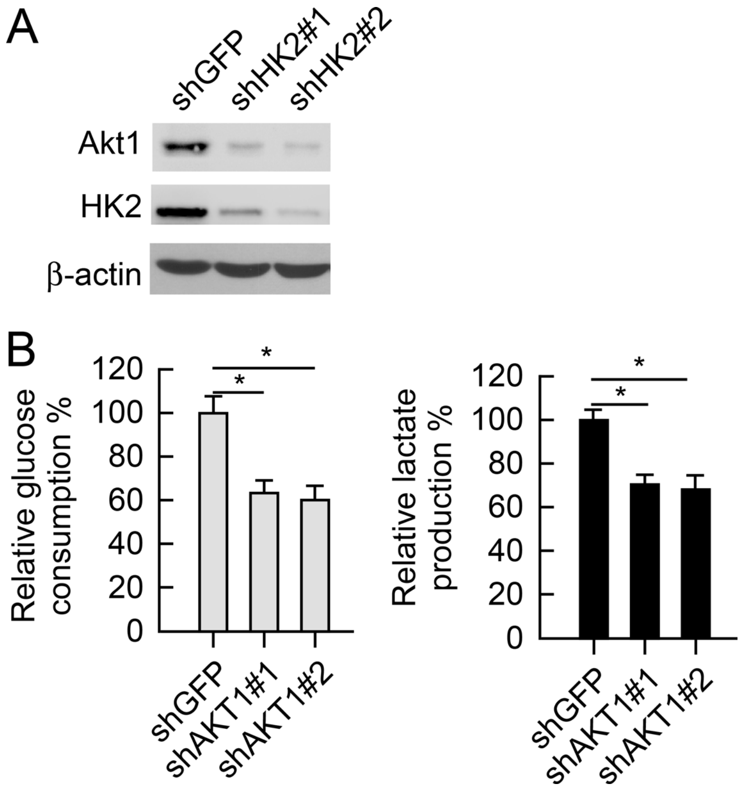

Supplementary Figure 3: Knockdown of Akt1 inhibits glycolysis. (**A**) western blot verifies knockdown of Akt1 in H460 cells. (**B**) the levels of glucose consumption (left) and lactate production (right) were examined in these cells. The asterisk indicates significant suppression (*p < 0.05) of glycolysis in Akt knockdown cells.

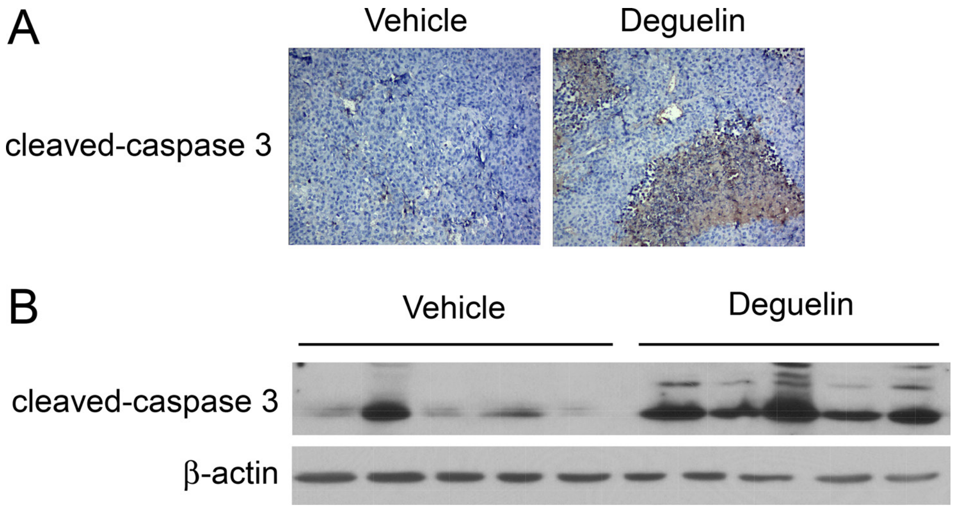

Supplementary Figure 4: (**A**) immunohistochemical staining examination of cleaved-caspase3 in tumor sections from vehicle group mice or deguelin-treated group mice. (**B**) western blot examination of cleaved-caspase3 in tumor sections from vehicle group mice or deguelin-treated group mice.
